# Two-year outcome of an observe-and-plan regimen for neovascular age-related macular degeneration treated with Aflibercept

**DOI:** 10.1007/s00417-017-3762-2

**Published:** 2017-08-10

**Authors:** Parmis Parvin, Marta Zola, Ali Dirani, Aude Ambresin, Irmela Mantel

**Affiliations:** Department of Ophthalmology, University of Lausanne, Jules Gonin Eye Hospital, Fondation Asile des Aveugles, Lausanne, Switzerland

**Keywords:** Age-related macular degeneration, Aflibercept, Anti-VEGF, Observe-and-plan regimen, Treat and extend regimen, Clinical burden, Chronic care management

## Abstract

**Purpose:**

The purpose of our study was to investigate the two-year outcome of Aflibercept treatment for neovascular age-related macular degeneration (nAMD), using the Observe-and-Plan regimen, an individually planned treatment regimen, based on the predictability of an individual’s need for retreatment, aiming to reduce the clinical burden.

**Methods:**

Our prospective study used the Observe-and-Plan regimen with Aflibercept to treat nAMD: Three loading doses, followed by monthly observation visits until the disease-recurrence interval was determined, which then was shortened by 2 weeks in a treatment plan for the next three injections without intermediate monitoring visits. The subsequent treatment plans were adjusted according to periodically assessed disease activity. The primary outcome measures were visual acuity changes, number of injections, and number of monitoring visits.

**Results:**

The study included 112 eyes of 102 patients with a mean age of 80.7 years (SD 7.6). Mean visual acuity (VA) improved from 61.8 ETDRS letters (20/60^+2^) at baseline, by 8.5, 8.0, and 6.2 letters at months 3, 12 and 24, respectively. Mean central retinal thickness was 438um at baseline, and reduced by 152um, 155um, and 150um at months 3, 12 and 24, respectively. The mean number of injections was 8.7 and 6.5 in the first and second year, respectively. The mean number of monitoring visits after baseline was 3.8 and 2.8 during the first and second year, respectively.

**Conclusions:**

The Observe-and-Plan regimen significantly improved VA, while fewer monitoring visits were needed as compared to other variable dosing regimens, thus reducing the workload for chronic care management of nAMD.

## Introduction

Age-related macular degeneration is a highly frequent macular pathology. Its natural course used to be the main reason of irreversible vision loss in individuals aged ≥50 years in industrialized countries. Since the introduction of intravitreal anti-vascular endothelial growth factor (anti-VEGF) treatment the proportion of legally blind eyes has been reduced. However, because monthly retreatment, as investigated by the pivotal trials for Ranibizumab [1, 2], places a heavy burden on the health care system and on patients, alternative regimens have been explored. The generalized reduction of treatment frequency with Ranibizumab to every 3 months resulted in the loss of initial visual acuity improvement. [3] For the introduction of Aflibercept, the corresponding pivotal trial investigated the option of fixed bimonthly retreatment, with success for an equal visual acuity outcome as with monthly retreatment, however, with a fluctuating pattern of structural outcome. [4].

The individual need for retreatment is highly variable between patients. [5] For some, monthly treatment is required, others do well on 3 monthly retreatment. [6] Generalized undertreatment is the great danger for functional outcome. [7] However, generalized overtreatment has been related to an increased risk of atrophic side effects [8, 9], beside the evident problem of exaggerated health care costs.

An individualized approach appears to be the most appropriate strategy, and the most widely used variable dosing regimen are the pro re nata (PRN) [10, 11] and treat and extend (TER) regimen. [12, 13] Frequent monitoring visits (monthly in PRN; with each injection in TER) are needed for these regimens in order to determine the individualized treatment need. For the health care institution, this remains a heavy burden due to the high number of patients.

Our group has evaluated the regularity and predictability of future treatment need in nAMD [14] which allowed for the development of an individually planned treatment regimen called Observe-and-Plan, reducing the number of monitoring visits needed. [15, 16] Given the satisfying results of this regimen using Ranibizumab, the aim of this study was to evaluate the results of the Observe-and-Plan regimen using Aflibercept. Functional results served as clinical validation of the regimen, but additional key outcomes were the number of injections and monitoring visits, or in other terms the factors which influence the work load.

## Materials and methods

This prospective study was undertaken in the medical retina department of a tertiary referral center (University Eye Hospital Jules Gonin in Lausanne, Switzerland). Informed consent was obtained from all individual participants included in the study.

### Study design

Our study involved a prospective noncomparative case series of nAMD patients, treatment naïve at baseline, undergoing intravitreal anti-VEGF treatment with Aflibercept, according to the treatment protocol of the Observe-and-Plan regimen. For 2 years the functional and anatomical results were recorded, along with the treatment time points, injection intervals, as well as the time points of the monitoring visits. The functional results served as clinical validation of the regimen as compared to the results in the literature of other regimens. The aim was to measure the number of monitoring visits and injections needed in order to obtain these functional results.

### Patient selection

Inclusion criteria were treatment-naïve nAMD with active subfoveal choroidal neovascularization (CNV) or retinal angiomatous proliferation (RAP), best corrected visual acuity (BCVA) between 20/25 and 20/400, a maximum lesion size of 12 disc areas and informed consent.

Exclusion criteria were atrophy or fibrosis in the center of the macula, any other macular pathology which might potentially interfere with the visual outcome, prior macular treatment, and poor image quality.

### Clinical investigation

Baseline examination and all monitoring visits included measurement of BCVA on the Early Treatment of Diabetic Study (ETDRS) chart, slit-lamp examination, measurement of intraocular pressure, dilated fundus examination, spectral domain optical coherence tomography (SD-OCT) on the Heidelberg Spectralis (6 mm, 49 lines; Heidelberg Engineering, Heidelberg, Germany) and fundus autofluorescence (Spectralis). Additional fluoresceine angiography (Topcon TRC-501X, Tokyo, Japan; or HRA, Heidelberg Engineering, Heidelberg, Germany) was performed at baseline, at month 3 and at month 24, completed with indocyanine green angiography (same machine) at baseline.

### Observe-and-plan regimen

**Fig. 1:**
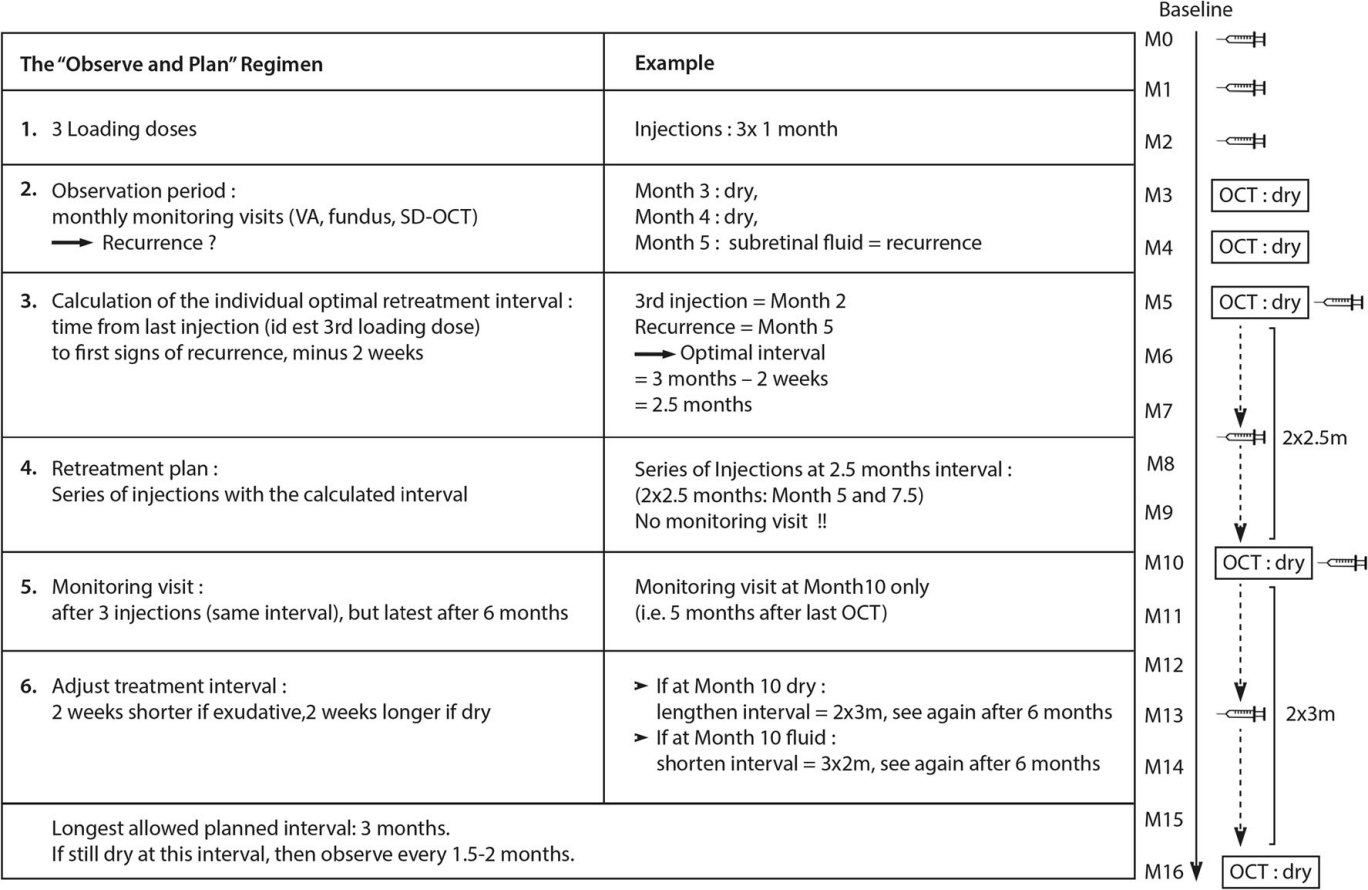
Description and illustration of the Observe-and-Plan regimen VA = visual acuity; SD-OCT = spectral domain optical coherence tomography; m = months

The principles of the Observe-and-Plan regimen are described in detail in a previous publication. [16] In summary, the regimen starts with treatment initiation, then measures the individual injection-recurrence interval, and finally applies a slightly shorter interval in a planned series of injections without intermediate monitoring visits. Over time, the interval is adjusted according to the results in periodical monitoring visits. In more detail, the regimen starts with three loading doses, followed by an observation period with monthly monitoring visits that allow for determining the injection-recurrence interval according to structural signs on SD-OCT examination (intra- or subretinal fluid) and/or fundus (hemorrhage). From the moment when disease activity reappears, the patient is retreated with an individualized but fixed treatment plan for several injections, which applies a 2 weeks shorter interval than the measured injection-recurrence interval (minimum 1 month, maximum 3 months). During the series of injections of the treatment plan, the patient is not monitored with BCVA or OCT, he directly presents to the injection procedure. Only after three planned injections the patient is seen in a monitoring visit that allows evaluating the applied interval and to adjust the subsequent treatment plans step by step, depending on the presence or absence of disease activity. However, this adjustment visit was no later than 6 months after the last monitoring visit, thus the possible treatment plans were 3 × 1 month, 3 × 1.5 months, 3 × 2 months, 2 × 2.5 months, 2 × 3 months. If still dry after 3 months, the patient was given the choice of continuing on a 3-month interval, or observation. The latter was performed every 1.5 months until 6 months after last injection and thereafter every 2 months. In case of recurrence later than 3 months since last injection, the per protocol treatment plan was 2 × 3 months. However, the investigator was encouraged to adjust to shorter intervals if needed according to his clinical appreciation. A graphical illustration with an example, helping to understand the regimen logic of observing, planning and adjusting, is given in Fig. 1. In addition, comprehensive flow charts have been previously published. [16, 17].

### Data analysis

The data from the monitoring visits and injection visits was collected for the corresponding time points. For statistical purposes, the visual acuity data and the central retinal thickness data from the SD-OCT were carried forward from last observation until next visit. This procedure was not repeated after a last visit in case of study drop-outs.

The main outcome measure was the mean BCVA change over time with an end point at 12 and 24 months. Additional visual outcomes were the proportion of eyes which lost ≥15 letters, which gained ≥0 letters and proportion of eyes which gained ≥15 letters. Further outcomes included the mean CRT change, treatment intervals during the first and second year, the number of injections and monitoring visits over 2 years.

## Results

A total of 112 eyes of 102 patients were included. The participants had a mean age of 80.7 years (SD 7.6) and included 72 women (70.6%). All patients were Caucasians. Ninety-seven patients (107 eyes) and 91 patients (99 eyes) completed the 12 and 24 months follow-up, respectively. Of the 11 patients that dropped out from the study, eight patients were lost from follow-up, two patients died during the study, and one patient discontinued following sterile endophthalmitis. Protocol violations with interrupted follow-up occurred with nine patients (nine eyes).

### Visual acuity outcomes

At baseline, mean best corrected visual acuity (BCVA) as measured on the ETDRS chart was 61.8 ETDRS letters (SD 15.4), corresponding to a Snellen equivalent of 20/60^+2^.

**Fig. 2:**
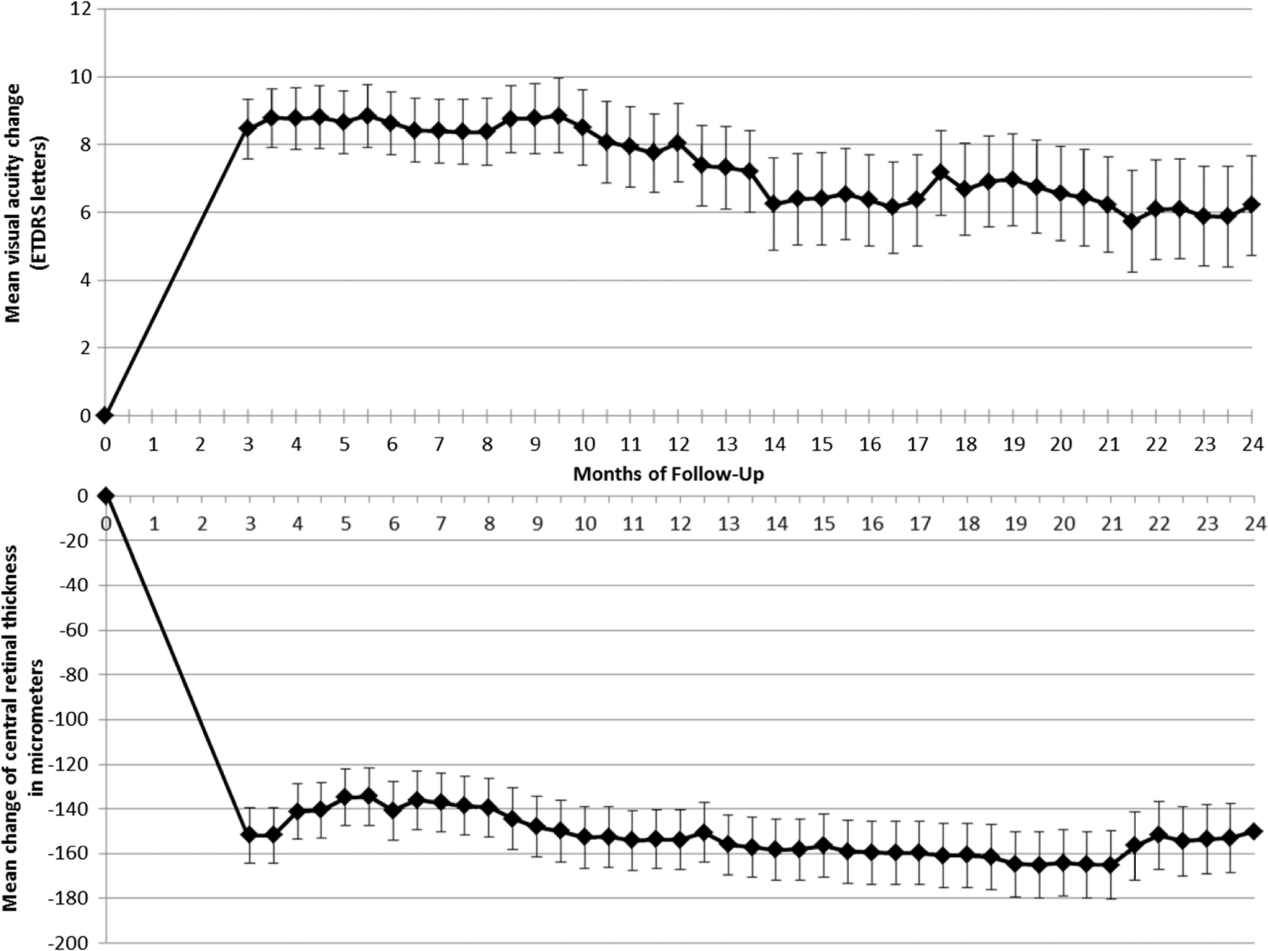
Mean visual acuity change and mean central retinal thickness change in study participants treated with intravitreal Aflibercept for neovascular age-related macular degeneration according to the Observe-and-Plan regimen during 2 years. Error bars represent the standard error

Under treatment, visual acuity improved by a mean of 8.5 (SD 9.2), 8.0 (SD 12.0), and 6.2 (SD 14.6) letters at Months 3, 12 and 24, respectively. All of these improvements are statistically significant (*P* < 0.001, paired t-test). Figure 2 shows the BCVA results graphically over time, using all half monthly time points.

Out of the 107 eyes which completed the first 12 months, the proportion of eyes that gained ≥15 letters, ≥ 0 letters, or that lost less than 15 letters was 26%, 80%, and 99%, respectively. At Month 24, the 99 eyes who completed this timepoint showed the corresponding proportions of 20%, 82%, and 92%, respectively.

### Structural outcome

The mean central retinal thickness measurements on SD-OCT improved from 438 μm (SD 148) at baseline by 152 μm, 154 μm, and 150 μm at Months 3, 12 and 24, respectively (Fig. 2).

### Factors impacting the clinical burden: Monitoring visits and injection procedures

**Fig. 3:**
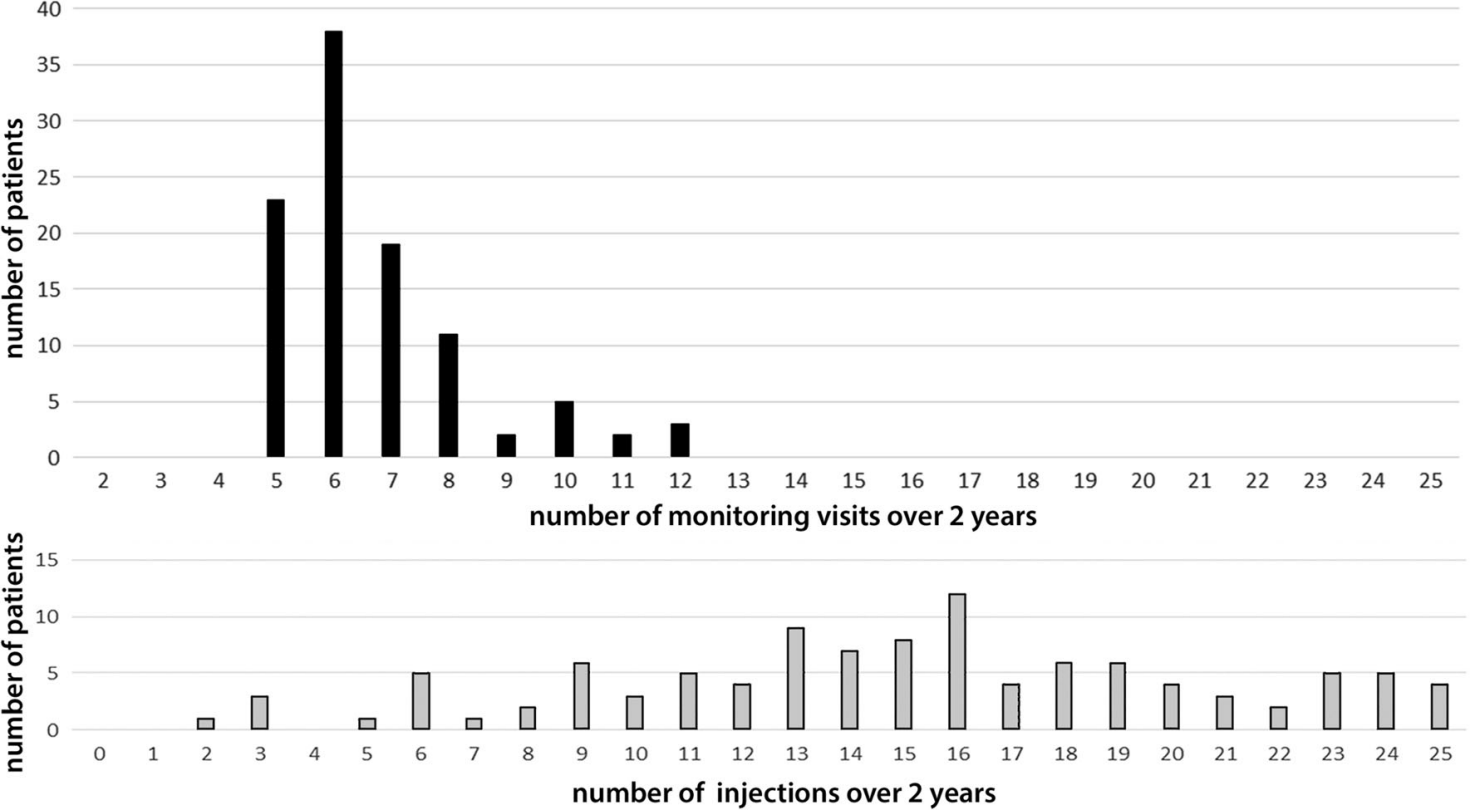
Distribution of the number of monitoring visits and the number of injections in patients treated with intravitreal Aflibercept for neovascular age-related macular degeneration according to the Observe-and-Plan regimen

The number of monitoring visits with ophthalmic examination after the baseline visit was a mean of 6.6 (SD 1.7) over the 2-year study duration (Fig. 3), out of which a mean of 3.8 (SD 1.0) monitoring visits were needed during the first year, and 2.9 (SD 1.2) during second year. The mean number of intravitreal injections of Aflibercept over 2 years was 15.3 (SD 5.2) including the first three loading doses. This divided into a mean of 8.7 (SD 3.0) and 6.3 (SD 6.2), in the first and second year, respectively. The distribution of the number of injections and monitoring visits is shown in Fig. 3. The graph reveals the shift of monitoring visits to lower numbers.

**Fig. 4:**
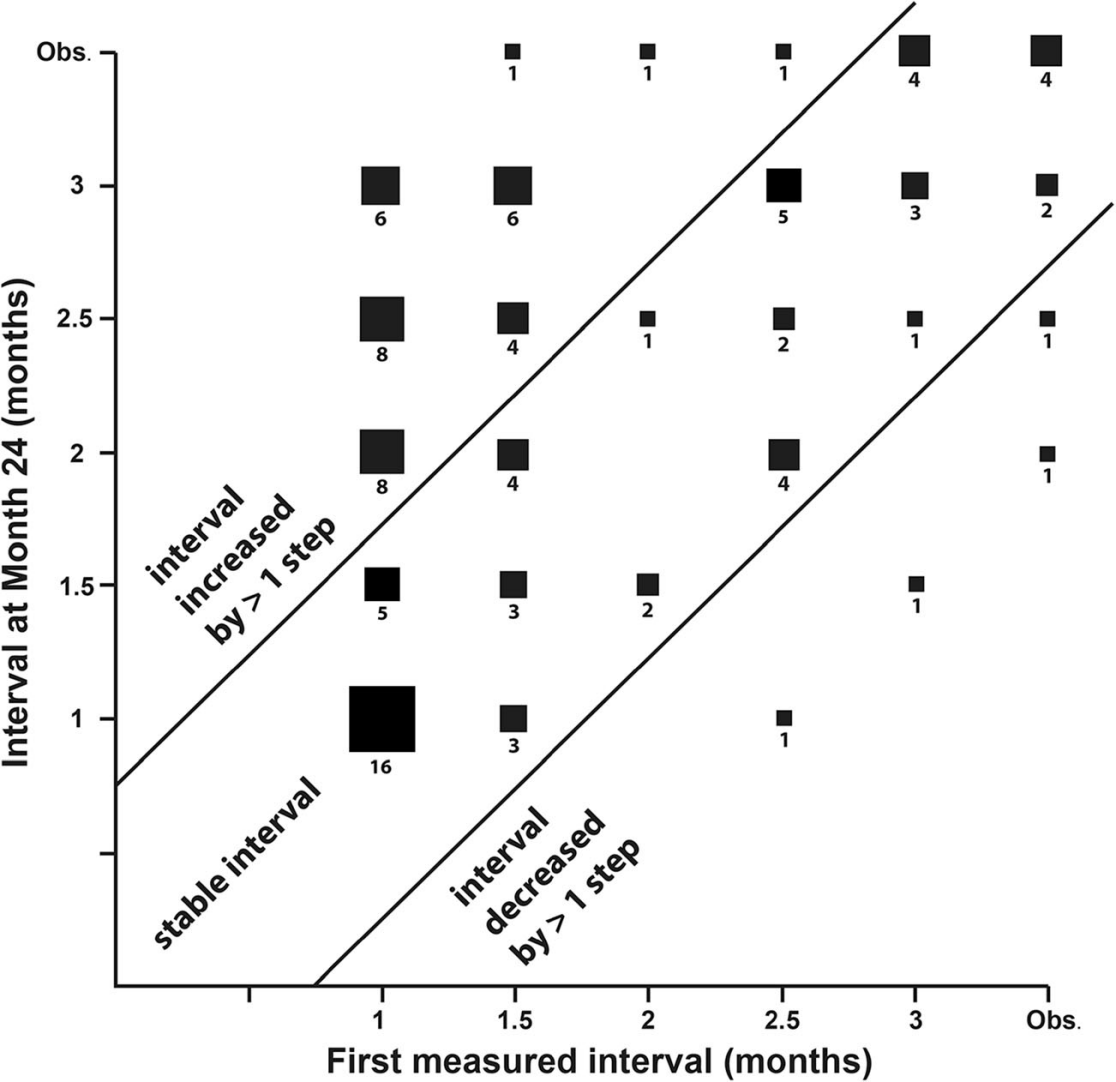
Distribution of the first measured interval after loading doses (horizontal axis), plotted against the last applied interval at Month 24 (vertical axis), for eyes that underwent treatment with Aflibercept for neovascular age-related macular degeneration according to the Observe-and-Plan regimen. The term “observation” is equivalent to any interval longer than 3 months. These eyes were followed regularly without planned injection

The mean retreatment interval as determined by the principles of the Observe-and-Plan regimen was 1.62 months between Month 3 and 12, and 1.90 months between Month 12 and 24. Table 1 summarizes the treatment intervals into categories and compares them for the first and second year: Short intervals up to 1.5 months (high treatment need), middle intervals between 1.5 and 3 months, and long treatment intervals of 3 months and more (low treatment need). The table shows the comparison between the first and the second year for 102 eyes with sufficient follow-up into year 2 (at least month 18). It reveals that the majority of eyes (58.8%) remained in their category, whether in the short interval category (23.5% of all), middle (21.6%) or long interval category (13.7%). A trend was found for a change to a category of longer intervals in the second year (35.3%); however, major changes were very rare (one single case from short interval to long intervals). On the other hand, a change into a shorter interval category in the second year was infrequent, observed in six eyes (5.9%), including only one eye with major change. An additional comparison was made using the very first and last interval measured, this is the interval after the loading doses of Aflibercept, and the interval at Month 24. The results are shown graphically in Fig. 4. It was found that 59 eyes (58%) remained within the stability criteria of +/− one step of interval change.

**Table 1:** Distribution of first year and second year treatment interval in categories of short, middle, and long treatment intervals according the the Observe-and-Plan regimen using Aflibercept for neovascular age-related macular degeneration

Treatment interval year 1		Treatment interval year 2			Total
		≤ 1.5 months	> 1.5 and <3 months	≥ 3 months	
≤ 1.5 months	N (% within the first year category)	**24 (46.2%)**	27 (51.9%)	1 (1.9%)	52 (100.0%)
	% of all patients	**23.5%**	26.5%	1.0%	51.0%
> 1.5 and	N (% within the first year category)	3 (9.1%)	**22 (66.7%)**	8 (24.2%)	33 (100.0%)
< 3 months	% of all patients	2.9%	**21.6%**	7.8%	32.4%
≥ 3 months	N (% within the first year category)	1 (5.9%)	2 (11.8%)	**14 (82.4%)**	17 (100.0%)
	% of all patients	1.0%	2.0%	**13.7%**	16.7%
	% of all patients	27.5%	50.0%	22.5%	100.0%

### Safety

In two eyes a pigment epithelium tear with major subretinal hemorrhage was observed. Both eyes showed severe BCVA loss (−48 letters and −74 ETDRS letters) despite continuation on monthly treatment.

Two eyes showed a complicated course due to severe intraocular inflammation after injection of Aflibercept. However, the vitreous tap remained sterile in both eyes, and their visual acuity recovered partially in one case (−16 ETDRS letters) and completely in the second case (+33 letters).

Two cases of death were considered unrelated to the study or the ocular treatment with Aflibercept.

Study-regimen related complications were not observed. However, in two patients (two eyes) the investigator decided to apply shorter intervals than normally required by the protocol, due to clinical impression of significant recurrence. In both eyes, visual acuity was well maintained during this recurrence and thereafter. All observed cases of vision loss >15 letters were attributable to regimen unrelated events such as fibrosis, atrophy, endophthalmitis, or pigment epithelium tear (see above).

## Discussion

Anti-VEGF treatment for nAMD represents a major challenge for clinical practice due to the high prevalence of the disease, the chronicity of the repetitive treatment, and the need for timely retreatment. The retreatment regimen plays an important role in order to respond to these challenges, and simultaneously adjust the treatment to the individual patient’s need. Several regimens have been introduced as an alternative to the fixed monthly or bimonthly retreatment schedule used in the pivotal trials. [1, 2] During the early years of anti-VEGF treatment, the pro re nata (PRN) regimen, based on monthly visits and re-injections as soon as signs of reactivation were discovered, [5, 10] was the most commonly adopted regimen in clinical routine. The downfall of this regimen is the impossibility to plan ahead, and the need for monthly visits. On this background, the Treat and Extend regimen offers the advantage to anticipate the next injection on an interval based concept, allowing for better ahead planning and a reduced number of visits. [12] In addition, it appears to better control for recurrences. [12] It is nowadays the most widely adopted regimen [ASRS survey 2015]. Simultaneously with the introduction of the Treat and Extend regimen, our group investigated the degree of regularity of injection-recurrence intervals. [14] The results revealed high intraindividual regularity (individual SD 0–2 weeks) and a good predictive value of the first measured interval after the loading doses (*R* = 0.70). [14] Based on these results, the Observe-and-Plan regimen was developed. Its efficacy and safety was reported, using Ranibizumab as the drug. [15, 16] The major advantages of the regimen were the planning ahead for up to 6 months and the reduced number of monitoring visits. This allowed for better clinical management of the human and technical resources. [18] The financial outcome in terms of cost-effectiveness was also reported in the same study [16], and was not repeated in the present report.

In the present study we intended to validate the Observe-and-Plan regimen for the use of Aflibercept. In addition, as a second study using the same regimen, it may serve as confirmation of the regimen validation. Truly, a regimen with strong reduction of the number of monitoring visits needs to demonstrate its reliability for functional outcomes. The results of this study revealed indeed a very similar outcome: Visual acuity was improved and this visual benefit was well maintained over two years, although with some minor loss over time. Some of this slow loss was attributable to a case of severe pigment epithelium rupture and major vision loss, a complication well known in cases of pigment epithelium detachment, with or without treatment. [19–21] We consider that the overall adequate functional results may serve as clinical validation of the regimen Observe-and-Plan. In addition, in comparison with the pivotal trials using Aflibercept for nAMD in a fixed monthly or bimonthly regimen during the first year, and pro re nata in the second year (VIEW studies) [22], our results are well comparable: After one and two years respectively, the VIEW studies reported a visual improvement of 8.3–9.3 and 6.6–7.6 ETDRS letters as compared to 8.0 and 6.2 ETDRS letters in our study. However, comparisons between studies are limited due to different inclusion criteria and methods.

With regard to adequateness of the regimen, the best possible maintenance of the mean visual acuity change over time is one of the most important outcome parameters. However, the concern clinicians may have when using the regimen Observe-and-Plan is the absence of intermediate monitoring visits. Some may fear potential undertreatment, resulting recurrences and subsequent visual loss. In order to address this question, we chose a double approach, in addition to the overall visual acuity results: First, we looked for recurrences which needed intensive treatment as decided by the investigator. Two cases were identified. However, there was no associated vision loss found. Second, we reviewed all cases with vision loss of >15 letters. In all of these cases, a clinical reason unrelated to the specific regimen was found (endophthalmitis, tear of the retinal pigment epithelium), and no case was associated with major exudative recurrence. Therefore, we conclude that the regimen is safe.

The satisfying functional results of the regimen were obtained with 15.3 injections over two years. This compares well with other variable dosing regimen, on the high end. The investigators actually applied a very strict retreatment policy in case of any intra- or subretinal fluid based on the very sensitive OCT examination on the Spectralis OCT, which might have led to early retreatment.

However, the main interest of the Observe-and-Plan regimen is the dramatically lower number of monitoring visits (3.8 in the first year, 2.9 in the second year) as compared to other retreatment regimens. The monitoring visits are in our experience the time consuming part for the health care team. The traditional variable dosing regimen PRN requires monthly monitoring visits. However, in real life the human and technical resources are frequently a limiting factor for the management of the high number of patients needing chronic care. This might explain some of the suboptimal outcome in real life reports. [7] In addition, monthly monitoring visits place a heavy burden on patients as well, potentially inducing compliance problems. The Observe-and-Plan regimen was developed with the perspective to optimize the number of visits without neglecting the individual need for treatment. The results of this study confirm that planning ahead for an individual’s need for treatment, while skipping monitoring visits, is possible and allows for good visual results. Thus, the regimen Observe-and-Plan reduces substantially the need for human and technical resources (by two thirds in comparison with the monthly PRN regimen, and approximately half in comparison with treat and extend). In an environment of limited human resources and/or limited access to the technical instruments such as OCT, this allows for better chronic care management of neovascular AMD care clinics with the given limited resources. In addition, patients did appreciate that the time spent in the clinic was reduced and well planned ahead.

Some limitations of the study need to be acknowledged: First, the absence of a direct comparison arm does not allow to formally prove non-inferiority. However, the analysis of BCVA improvement and stability over time is an indirect evidence of the value of the regimen. Second, the number of participating patients was relatively limited. Third, the number of protocol violations and the number of patients which were lost to follow-up were not low and could have influenced the results. However, we were unable to find a trend for different outcome for these patients. Fourth, it would have been interesting to compare the outcome of this study with the previous analog study using Ranibizumab. Unfortunately, this was not possible. In addition to the fact that independent studies are never perfectly comparable, the use of different OCT machines and a different team of investigators make the direct comparison impossible. However, the results are overall very similar and not suggestive of major differences. In particular, no sign of lower need for retreatment with Aflibercept as compared with Ranibizumab was found.

In conclusion, the Observe-and-Plan regimen is a promising approach in order to treat nAMD eyes with an adequate number of anti-VEGF injections, individualized according to their need, yet planned ahead avoiding unnecessary monitoring visits. This allows for good visual outcome, with the usual mean number of injections, but with the major advantage of lower need for resources due to dramatically reduced number of monitoring visits.

## References

[1] Rosenfeld PJ, Brown DM, Heier JS, Boyer DS, Kaiser PK, Chung CY, Kim RY (2006). Ranibizumab for neovascular age-related macular degeneration. NEngl J Med.

[2] Brown DM, Kaiser PK, Michels M, Soubrane G, Heier JS, Kim RY, Sy JP, Schneider S (2006). Ranibizumab versus verteporfin for neovascular age-related macular degeneration. NEngl J Med.

[3] Regillo CD, Brown DM, Abraham P, Yue H, Ianchulev T, Schneider S, Shams N (2008). Randomized, double-masked, sham-controlled trial of ranibizumab for neovascular age-related macular degeneration: PIER study year 1. Am J Ophthalmol.

[4] Heier JS, Brown DM, Chong V, Korobelnik JF, Kaiser PK, Nguyen QD, Kirchhof B, Ho A, Ogura Y, Yancopoulos GD, Stahl N, Vitti R, Berliner AJ, Soo Y, Anderesi M, Groetzbach G, Sommerauer B, Sandbrink R, Simader C, Schmidt-Erfurth U, View GVS (2012). Intravitreal aflibercept (VEGF trap-eye) in wet age-related macular degeneration. Ophthalmology.

[5] Lalwani GA, Rosenfeld PJ, Fung AE, Dubovy SR, Michels S, Feuer W, Davis JL, Flynn HW, Esquiabro M (2009). A variable-dosing regimen with intravitreal ranibizumab for neovascular age-related macular degeneration: Year 2 of the PrONTO study. Am J Ophthalmol.

[6] Schmidt-Erfurth U, Eldem B, Guymer R, Korobelnik JF, Schlingemann RO, Xer-Siegel R, Wiedemann P, Simader C, Gekkieva M, Weichselberger A (2011). Efficacy and safety of monthly versus quarterly ranibizumab treatment in neovascular age-related macular degeneration: The EXCITE study. Ophthalmology.

[7] Holz FG, Tadayoni R, Beatty S, Berger A, Cereda MG, Hykin P, Staurenghi G, Wittrup-Jensen K, Altemark A, Nilsson J, Kim K, Sivaprasad S (2016). Key drivers of visual acuity gains in neovascular age-related macular degeneration in real life: Findings from the AURA study. Br J Ophthalmol.

[8] Chakravarthy U, Harding SP, Rogers CA, Downes SM, Lotery AJ, Culliford LA, Reeves BC, investigators Is (2013). Alternative treatments to inhibit VEGF in age-related choroidal neovascularisation: 2-year findings of the IVAN randomised controlled trial. Lancet.

[9] Grunwald JE, Daniel E, Huang J, Ying GS, Maguire MG, Toth CA, Jaffe GJ, Fine SL, Blodi B, Klein ML, Martin AA, Hagstrom SA, Martin DF, Group CR (2014). Risk of geographic atrophy in the comparison of age-related macular degeneration treatments trials. Ophthalmology.

[10] Fung AE, Lalwani GA, Rosenfeld PJ, Dubovy SR, Michels S, Feuer WJ, Puliafito CA, Davis JL, Flynn HW, Esquiabro M (2007). An optical coherence tomography-guided, variable dosing regimen with intravitreal ranibizumab (Lucentis) for neovascular age-related macular degeneration. AmJ Ophthalmol.

[11] Martin DF, Maguire MG, Ying GS, Grunwald JE, Fine SL, Jaffe GJ (2011). Ranibizumab and bevacizumab for neovascular age-related macular degeneration. NEngl J Med.

[12] Gupta OP, Shienbaum G, Patel AH, Fecarotta C, Kaiser RS, Regillo CD (2010). A treat and extend regimen using ranibizumab for neovascular age-related macular degeneration clinical and economic impact. Ophthalmology.

[13] Wykoff CC, Croft DE, Brown DM, Wang R, Payne JF, Clark L, Abdelfattah NS, Sadda SR, Group T-AS (2015). Prospective trial of treat-and-extend versus monthly dosing for Neovascular age-related macular degeneration: TREX-AMD 1-year results. Ophthalmology.

[14] Mantel I, Deli A, Iglesias K, Ambresin A (2013). Prospective study evaluating the predictability of need for retreatment with intravitreal ranibizumab for age-related macular degeneration. Graefe's archive for clinical and experimental ophthalmology = Albrecht von Graefes Archiv fur klinische und experimentelle Ophthalmologie.

[15] Gianniou C, Dirani A, Ferrini W, Marchionno L, Decugis D, Deli A, Ambresin A, Mantel I (2015). Two-year outcome of an observe-and-plan regimen for neovascular age-related macular degeneration: How to alleviate the clinical burden with maintained functional results. Eye (London, England).

[16] Mantel I, Niderprim SA, Gianniou C, Deli A, Ambresin A (2014). Reducing the clinical burden of ranibizumab treatment for neovascular age-related macular degeneration using an individually planned regimen. Br J Ophthalmol.

[17] Gianniou C, Dirani A, Ferrini W, Marchionno L, Decugis D, Deli A, Ambresin A, Mantel I (2015). Two-year outcome of an observe-and-plan regimen for neovascular age-related macular degeneration: How to alleviate the clinical burden with maintained functional results. Eye (London, England).

[18] Mantel I (2015). Optimizing the anti-VEGF treatment strategy for Neovascular age-related macular degeneration: From clinical trials to real-life requirements. Translational vision science & technology.

[19] Cho HJ, Kim KM, Kim HS, Lee DW, Kim CG, Kim JW (2016). Response of pigment epithelial detachment to anti-vascular endothelial growth factor treatment in age-related macular degeneration. Am J Ophthalmol.

[20] Chan CK, Meyer CH, Gross JG, Abraham P, Nuthi AS, Kokame GT, Lin SG, Rauser ME, Kaiser PK (2007). Retinal pigment epithelial tears after intravitreal bevacizumab injection for neovascular age-related macular degeneration. Retina.

[21] Weinberger AW, Thiel M, Mohammadi B, Theofylaktopoulos I, Thumann G, Walter P (2007). Retinal pigment epithelium tears after intravitreal bevacizumab in pigment epithelium detachment. Am J Ophthalmol.

[22] Schmidt-Erfurth U, Kaiser PK, Korobelnik JF, Brown DM, Chong V, Nguyen QD, Ho AC, Ogura Y, Simader C, Jaffe GJ, Slakter JS, Yancopoulos GD, Stahl N, Vitti R, Berliner AJ, Soo Y, Anderesi M, Sowade O, Zeitz O, Norenberg C, Sandbrink R, Heier JS (2014). Intravitreal aflibercept injection for neovascular age-related macular degeneration: Ninety-six-week results of the VIEW studies. Ophthalmology.

